# Supplementary material for: Cloud BioLinux: pre-configured and on-demand bioinformatics computing for the genomics community
Source: BMC Bioinformatics. 2012 Mar 19;13:42. doi: 10.1186/1471-2105-13-42 (PMC3372431; doi:10.1186/1471-2105-13-42)
Supplement: Additional file 1 — Supplementary 1 Cloud BioLinux software documentation in the form of a mini, self-contained website. Users need to download and uncompress the .zip file, and open through a web browser the "index.html" file available on the main directory. (ZIP 1823 kb). [file 1471-2105-13-42-S1.ZIP › Cloud-BioLinux-Package-Documentation/docs/mesquite.html]

Bio-Linux Software Documentation Pages

Back to search form

## mesquite

|  |  |
| --- | --- |
| Name | mesquite |
| Description | **Mesquite** is software for evolutionary biology, designed to help biologists analyze comparative data about organisms. Its emphasis is on phylogenetic analysis, but some of its modules concern population genetics, while others do non-phylogenetic multivariate analysis. Because it is modular, the analyses available depend on the modules installed.  Analyses include:   - Reconstruction of ancestral states (parsimony, likelihood) - Tests of process of character evolution, including correlation - Simulation of character evolution (categorical, DNA, continuous) - Parametric bootstrapping (integration with programs such as PAUP\* and NONA)     - Morphometrics (PCA, CVA, geometric morphometrics) - Coalescence (simulations, other calculations) - Tree comparisons and simulations (tree similarity, Markov speciation models) |
| Homepage | http://mesquiteproject.org/mesquite/mesquite.html |
| Remote Documentation | http://mesquiteproject.org/Mesquite\_Folder/docs/mesquite/manual.html |
